# Exploring sociodemographic factors and coping strategies before and during the COVID-19 pandemic among paramedics working in a Middle Eastern country

**DOI:** 10.5339/qmj.2026.58

**Published:** 2026-09-01

**Authors:** Mohammed Jameel Al Barbari, Padarath Gangaram, Hmoud Alolimat, Gary Kenward, James Laughton, Guillaume Alinier

**Affiliations:** 1 Department of Social Work, Doha Institute for Graduate Studies, Doha, Qatar; 2 Hamad Medical Corporation Ambulance Service, Doha, Qatar; 3 Faculty of Health Sciences, Durban University of Technology, Durban, South Africa; 4 Weill Cornell Medicine-Qatar, Doha, Qatar; 5 School of Health, Medicine, and Life Sciences; University of Hertfordshire, Hatfield, HERTS, UK; 6 Faculty of Health and Life Sciences, Northumbria University, Newcastle upon Tyne, UK

**Keywords:** Coping strategies, paramedics, COVID-19, mental health, Qatar

## Abstract

**Background:**

Paramedics often work under pressure, making them vulnerable to burnout and depression, but the Coronavirus disease (COVID-19) pandemic was an unprecedented crisis in modern times. This study examines the coping strategies employed by paramedics in Qatar’s national Ambulance Service prior to and during the COVID-19 pandemic.

**Methods:**

In early 2021, we conducted an anonymous online survey using a modified version of Carver’s Brief COPE scale, a validated tool measuring coping mechanisms. The survey was shared with 1,100 paramedics.

**Results:**

The data from 272 valid responses revealed a drop in several healthy coping strategies during the pandemic, including meditation, social activities, physical exercise, and religious practices. Instead, the use of comfort food as a coping method increased significantly. A paired *t*-test showed that the average coping strategy score decreased significantly from 48.95 (± 6.78) before the pandemic to 45.15 (± 6.7) during the pandemic (*p* < 0.05) out of 85 points. Interestingly, behaviours like self-reported smoking and substance use remained unchanged (*p* > 0.05).

**Conclusion:**

The decline in helpful coping behaviours during the pandemic likely reflects the elevated stress and limited opportunities for self-care due to lockdowns and work demands. These findings highlight an urgent need for better mental health support and targeted interventions to strengthen paramedics’ resilience, both during crises and in their everyday roles.

## 1. INTRODUCTION

When the Coronavirus disease (COVID-19) pandemic emerged in late 2019, few could have predicted how quickly it would upend people’s daily lives globally.^1^ For many, the pandemic brought fear and uncertainty, but for frontline healthcare workers, especially paramedics, it brought an entirely new level of psychological and physical strain. They were not just witnessing the crisis unfold; they were standing at its epicentre, often the first to care for the sickest patients and the last to find rest.

Even before the pandemic, being a paramedic meant managing extreme pressure responding to emergencies, witnessing trauma, and making split-second decisions under pressure.^2^ During COVID-19, the stress multiplied. Infection risks, emotional fatigue, and the overwhelming workload became constant.^3^ Despite these challenges, many paramedics continued to serve with courage and resilience, drawing on personal strengths like adaptability, emotional regulation, and even humour to cope with the situation.

Globally, studies confirmed what many on the frontlines already knew and experienced: burnout was rampant. In India, nearly half of healthcare workers reported personal burnout, with more than a quarter citing work-related exhaustion.^4^ In China, anxiety, depression, and post-traumatic stress disorder (PTSD) symptoms were widespread among hospital staff treating COVID-19 cases.^5^ Emergency Medical Services (EMS) personnel, in particular, were hit hard. About 20% of paramedics were believed to have experienced PTSD at a rate notably higher than that of other healthcare providers.^6^

Fatigue, long shifts, and disrupted sleep further worsened their mental and physical well-being. As Patterson et al.^7^ noted, many paramedics experience significant exhaustion, which not only affects their health but also increases the risk of errors and reduces their performance. However, not every paramedic responded to the pandemic crisis in the same way. Some struggled deeply, while others found ways to persevere, leaning on coping mechanisms that helped them stay grounded.^8^ Interestingly, research suggests that some paramedics reported lower stress during the pandemic, a finding that is surprising.^3^ However, this could point to the remarkable psychological resources these individuals possess: resilience, emotional strength, and the ability to adapt in the face of adversity.^9,10^ Understanding and reinforcing these coping strategies could be the key to sustaining a healthier, more resilient paramedic workforce in the future.

This study seeks to address what factors (e.g., age, education, marital status, etc.) could have influenced how paramedics coped with the pandemic by focusing on the paramedics at Hamad Medical Corporation Ambulance Service (HMCAS) in Qatar. It is important to note that the EMS population studied is almost entirely constituted of expatriates. HMCAS is a very well-resourced and organised prehospital care provider responding to all health-related emergency calls on a free-for-all service and irrespective of the patients’ residency status.^11^ HMCAS played a key role during the pandemic, such as managing drive-through testing centres and isolation facilities and transporting acutely ill patients.^12–14^ This study explores how paramedics managed stress before and during the pandemic, and whether their coping strategies changed over time. By examining the role of demographic and social factors, the study hopes to offer insights into how EMS systems can better support their staff not just during global emergencies, but in the daily pressures of prehospital care.

## 2. METHODS

### 2.1. Study design

A quantitative design was adopted, using an anonymous online survey as the primary data collection tool to promote truthful responses. The survey incorporated validated instruments adapted to the prehospital care context and the pandemic.

### 2.2. Study population

Ambulance Paramedics (APs) at Hamad Medical Corporation Ambulance Service (HMCAS) are primarily responsible for responding to emergency 999 calls, providing prehospital assessment, basic and advanced life support, and transporting patients to appropriate healthcare facilities. Critical Care Paramedics (CCPs) have a higher level of scope of practice and can manage high-acuity patients requiring enhanced interventions such as advanced airway management, mechanical ventilation, complex pharmacological therapy, and interfacility critical care transfers.^11^

### 2.3. Sampling approach and sample size

A purposive sampling strategy was used to target APs and CCPs with frontline experience prior to the onset of the COVID-19 pandemic. Based on Geiger’s equation and aiming for a 95% confidence level, the minimum required sample size was calculated as 285 participants. The survey was distributed to all registered paramedics employed by HMCAS at the time of the study (*N* = 1,100).

Participants were required to have been employed at HMCAS prior to 1 December 2019, to have pre-pandemic prehospital care experience, to be actively responding to 999 emergency calls, and to work at least two clinical shifts per month. Non-frontline healthcare workers and paramedics who joined HMCAS after December 2019 were excluded.

### 2.4. Data collection

Data were collected in early 2021 via an online survey which included participants’ scope of practice, gender, ethnicity, nationality, age, duration of employment with HMCAS, total years of clinical experience, education level, marital status, spouse living status in Qatar, and previous COVID-19 infection.

Coping strategies were assessed using a modified version of the Brief COPE scale developed by Carver.^15^ The instrument was minimally adapted to reflect the context of the COVID-19 pandemic and the operational realities experienced by frontline paramedics. The primary modification involved incorporating references to “COVID-19” within selected survey items to improve contextual relevance while preserving the original coping constructs and structure of the validated instrument.

Prior to full distribution, the questionnaire underwent expert review to assess face and content validity. Pilot testing was also conducted with a small group of paramedics to evaluate the clarity, comprehensibility, and relevance of the survey items. Minor wording refinements were subsequently implemented based on participants’ feedback.

The survey included 17 questions: 16 closed-ended items rated on a 5-point Likert scale (1 = Never to 5 = Always, range of 16 to 80 points), and one open-ended “Other” item allowing participants to specify additional coping strategies. The questions captured self-reported behaviours before and during the COVID-19 pandemic.

The survey was distributed via SMS to all registered paramedics (*N* = 1,100). Although the minimum required sample size was calculated as 285 to achieve a 95% confidence level, a total of 272 valid responses were included in the final analysis, representing a minor shortfall. However, the achieved sample remained adequate for statistical analysis and inference.

### 2.5. Data analysis

Data were analysed using Statistical Package for Social Sciences (SPSS) version 25. Descriptive statistics were used to summarize demographic characteristics. Data distribution was assessed for normality prior to analysis. Associations between variables were explored using cross-tabulations and chi-square tests, while paired *t*-tests were conducted to compare coping strategies before and during the pandemic. Where applicable, eta squared (η²), a measure of effect size, was calculated to assess the influence of sociodemographic variables on coping behaviours.

## 3. RESULTS

A total of 272 paramedics who responded to the survey met the inclusion criteria and were included in the final analysis. Although this number was slightly below the calculated minimum sample size, the achieved sample was considered sufficient for statistical analysis and inference. The participants were predominantly male (94.9%), with the majority aged between 31 and 40 years (68%). 90% of the paramedics had more than six years of experience and had an undergraduate qualification (Table 1). The various demographic characteristics of the participants were representative of the overall paramedic population.

### 3.1. Coping strategies before and during the pandemic

The analysis of coping strategies among paramedics revealed statistically significant differences between the pre-pandemic period and during the COVID-19 pandemic. Specifically, there was a reduction in the use of coping behaviours, such as meditation, attending the cinema, watching television, socialising with friends and family, engaging in both individual and team sports, and participating in religious practices. In contrast, a small increase was observed in the consumption of comfort food as a coping strategy. However, the use of substances, including cigarettes, shisha, alcohol, and recreational drugs, did not show any statistically significant change (*p* > 0.05). Overall, the total coping strategies score decreased significantly from a pre-pandemic mean of 48.96 to 45.18 out of 80 (*p* < 0.001), indicating a reduction in reported coping engagement during a period of increased occupational and psychological demands (Table 2). Participants also reported additional coping mechanisms, including leisure activities, passive behaviours, and work-related adjustments (Table 3).

### 3.2. Coping levels

During the pandemic, paramedics’ self-reported coping levels changed significantly (McNemar-Bowker test, *p* < 0.001). Before the pandemic, most participants (76.1%) reported high coping levels, but during the pandemic, the proportion dropped to 54.4% (Table 4).

### 3.3. Association between different demographic factors and coping scores

APs showed a statistically significant decrease in coping scores during the pandemic, whereas CCPs demonstrated a smaller, non-significant decrease. However, independent *t*-tests revealed no statistically significant differences between the two groups in overall coping scores either before or during the pandemic, suggesting that clinical role alone did not explain differences in coping levels.

Gender-related findings revealed statistically significant within-gender declines in coping scores over time (Table 5). Although coping scores decreased in both genders, the difference in pre-pandemic scores was not statistically significant (*p* = 0.057). However, during the pandemic, female paramedics reported significantly higher coping scores than their male counterparts (*p* = 0.025).

When comparing findings by ethnic group, Southeast Asian paramedics exhibited the most substantial decline in coping scores, with a mean difference of −5.07, followed by Caucasian paramedics (−4.77) and South Asian paramedics (−3.36) (Table 5). Conversely, Black/African paramedics did not experience a statistically significant change; however, the small sample size limits the generalizability of this finding. When analysed by nationality, statistically significant reductions in coping scores were observed across all groups, particularly among Indian, Tunisian, and Filipino paramedics. However, no statistically significant between-nationality differences were found in coping scores either before or during the pandemic (Table 5).

A significant decline in coping scores was observed across all age groups during the pandemic (*p* < 0.05) (Table 5). The most pronounced reduction occurred among paramedics aged 31–40 years, with a mean decrease of 4.18 points. The 51–60-year age group showed the smallest decline (-2.00), although this group also reported the lowest baseline coping scores prior to the pandemic. In terms of between-group comparisons, significant differences were evident before the pandemic (*p* < 0.001), with older paramedics (51–60 years) reporting significantly lower coping scores than younger colleagues. These disparities persisted during the pandemic (*p* = 0.018), with the oldest group continuing to demonstrate the lowest coping levels (Table 5).

A significant reduction in coping scores was also observed across all categories of duration of employment at HMCAS (*p* < 0.001) (Table 5). The greatest decline was reported among paramedics with more than 10 years of employment (mean difference = −3.90), followed closely by those with 7–10 years (−3.87). Despite these within-group reductions, no statistically significant differences were observed between employment duration groups either before or during the pandemic (Table 5).

Significant reductions in coping scores were observed across all experience groups during the pandemic (*p* < 0.05) (Table 6), the most pronounced being among paramedics with 11–15 years of experience (−4.44). Despite this, no statistically significant differences in coping scores were found between experience groups before or during the pandemic.

Paramedics in all educational qualification groups also had significant reductions in coping scores during the pandemic (*p* < 0.05) (Table 6). The most substantial decline was among those with a bachelor’s degree (−4.01), and the smallest decline was for master’s or PhD graduates (−2.72). While no statistically significant differences in coping scores were found between educational qualification groups before the pandemic, the differences that emerged during the pandemic were statistically significant (*p* = 0.032). Specifically, paramedics with a Diploma or lower qualifications reported significantly lower coping scores than their colleagues with a higher qualification.

Statistically significant reductions in coping scores were observed among both married and single paramedics during the pandemic (Table 6). Divorced or separated paramedics experienced a steep decline, but it was not statistically significant, probably due to the very small subgroup sample size. Despite within-group changes, no statistically significant differences in coping scores were observed. Irrespective of their spouse’s location, all paramedics experienced a statistically significant and noticeable decline in coping scores during the pandemic (Table 6). Paramedics with their spouses in Qatar experienced a smaller drop in coping scores compared to those with spouses outside Qatar.

Whether paramedics tested positive for COVID-19 or remained negative during the pandemic, they all showed statistically significant declines in coping scores during the pandemic (Table 6).

## 4. DISCUSSION

During the COVID-19 pandemic, Qatar implemented a centralized public health response emphasizing rapid testing, contact tracing, quarantine services, and early vaccination. These measures were key to reducing disease severity and transmission.^16^ By May 2023, when the World Health Organization (WHO) declared that COVID-19 was no longer a global health emergency, millions of cases and deaths had been reported worldwide.^17^ Qatar, however, experienced one of the lowest COVID-19 fatality rates globally, with an all-cause mortality of 0.96 per 1,000 person-years and a COVID-19-specific mortality of 0.13 per 1,000 person-years.^18^ Factors such as a relatively young population, strong healthcare infrastructures, and early vaccine rollout likely contributed to these outcomes.^16,18^

Despite this success, frontline healthcare workers, including paramedics, faced significant psychological distress due to heavy workload demands, uncertainty, and emotional strain from caring for patients. Related work conducted by our team highlighted that Qatar’s paramedics experienced elevated stress and post-traumatic stress symptoms during the pandemic, underscoring the urgent need for targeted mental health interventions.^19^

Adaptive coping behaviours among paramedics declined significantly during the pandemic (Table 2). Reductions were noted in reported physical activity, meditation, social interactions, and religious practices. Lockdowns, restricted gatherings, closure of gyms, and operational pressures limited opportunities for routine stress-relief activities. Religious coping showed a dual effect. Positive religious practices, such as prayer and trust in God, provided emotional relief, whereas negative religious coping (e.g., feelings of divine punishment) was linked to heightened distress.^20,21^ Maladaptive strategies, such as slightly increased consumption of comfort food, reflected heightened anxiety and stress-driven behaviours.^22,23^ These findings align with global literature demonstrating that pandemic-related restrictions disrupted coping routines and increased psychological burden among healthcare workers.^24,25^

Despite individual variations, the overall trend across all paramedics was a decline in coping scores during the pandemic (Tables 5 and 6). Workload, age, clinical role, gender, and education influenced their coping level to some extent, yet pandemic-induced stressors, uncertainty, high patient load, disrupted routines, and emotional strain universally reduced resilience.

Female paramedics maintained slightly higher coping scores than males (Table 5). This is consistent with prior research suggesting women often employ more proactive coping strategies, such as seeking social support or emotional expression, which may buffer psychological stress.^22^ Female paramedics represented a small proportion of the study sample (approximately 5%), which limits the generalizability of gender-related findings and should be interpreted with caution.

Older paramedics exhibited the lowest coping scores (Table 5), possibly due to higher health risks, limited digital literacy for online coping tools access, or compounded family responsibilities. However, the largest subgroup (31–40 years) also experienced substantial declines, indicating that younger paramedics were not immune to pandemic-induced stress, and emphasizing the need for age-sensitive mental health strategies.^26,27^

Lower educational attainment was associated with poorer coping outcomes (Table 6). Higher qualifications may provide enhanced problem-solving skills, confidence, and resilience, suggesting that paramedics with diploma-level education or lower may benefit from tailored peer support and educational programs.^28,29^

While differences between APs and CCPs were not statistically significant, APs had statistically significant and slightly larger reductions in coping scores (Table 5). This may indicate that systemic factors, such as resource availability, organizational culture, and cumulative exposure, play a stronger role in influencing coping than clinical designation alone.^30,31^

Mid-career paramedics with 11–15 years of experience showed the largest decreases in coping (Table 6), likely due to the combined stress of clinical duties and personal responsibilities.

All ethnic and nationality groups experienced declines in coping scores, with no statistically significant between-group differences (Table 5). This indicates that pandemic stressors had a broadly universal impact, regardless of ethnic or nationality background. Limited sample sizes for smaller subgroups, such as Black/African paramedics, may have constrained statistical power and interpretation.

The study population was culturally diverse, with 54.8% identifying as Arabian (Table 5). A high proportion of participants were married (88.2%), and most (90.1%) reported that their spouses were living in Qatar, while only 9.9% had spouses residing abroad (Table 1). Greater declines in coping scores were observed among married paramedics and those separated from spouses (Table 6), possibly due to added family responsibilities or emotional strain.

Interestingly, despite their frontline roles, 75% of participants reported not contracting COVID-19, reflecting effective infection control measures and personal precautions. A separate study conducted with HMCAS paramedics determined that they had a good knowledge about COVID-19 and a positive attitude to infection control practices.^32^

Notably, reductions in coping occurred regardless of paramedics’ COVID-19 infection status (Table 6), demonstrating that stress was driven more by the broader operational and psychosocial challenges of the pandemic than personal illness.

Religion emerged as both a protective and a risk factor in psychological coping. In devout societies like Qatar, the disruption of religious routines, especially communal rituals like Friday prayers, had a tangible emotional impact. This finding is consistent with studies in Saudi Arabia^28^, Morocco^21^, and Tunisia^33^, all of which highlighted both the benefits and psychological hazards of religious interpretations during crises. For instance, in Saudi Arabia, prayer and Qur’anic recitation were among the most utilized coping tools. However, in Tunisia, negative religious beliefs (e.g., seeing COVID-19 as divine punishment) correlated with worsened mental health. Similarly, a review underscored that maladaptive religious coping was a consistent global risk factor for mental health deterioration.^34^ These insights call for culturally tailored interventions that integrate positive religious practices into psychological support. Initiatives such as online prayer groups, spiritual counselling, and faith-based mindfulness sessions could restore spiritual resilience. Collaboration with religious leaders to reinforce Islamic concepts, such as Tawakkul (trust in God) and Sabr (patience), may help reframe distress and foster hope among frontline workers.

Despite individual variations, the overall trend across all HMCAS paramedics was a decline in coping scores during the pandemic (Table 5). Workload, age, clinical role, gender, and education influenced coping to some extent, yet pandemic-induced stressors uncertainty, high patient load, disrupted routines, and emotional strain universally reduced resilience. Notably, reductions in coping occurred regardless of paramedics’ COVID-19 infection status, demonstrating that stress was driven more by the broader operational and psychosocial challenges of the pandemic than personal illness.

These findings are consistent with international studies showing widespread burnout and emotional exhaustion among healthcare workers, irrespective of direct patient exposure or demographic factors.^31^ Organizationally, wellness programs, open discussions about the pandemic’s impact, flexible scheduling promoting work-life balance, and recreational spaces could help restore access to healthy coping avenues. Further education and workshops would empower paramedics to develop essential skills and adaptive mechanisms, ensuring they are more equipped and prepared to work under pressure and in the event of possible health crises, fostering a more resilient and capable workforce. On a systemic level, universal access to mental health services, including confidential counselling and psychological first aid, should be standard during crises.

### 4.1. Strengths and limitations

This study contributes to the body of research on how paramedics cope with stress during a pandemic, particularly within the often-overlooked Middle Eastern context. The culturally diverse sample enhances the strength of the findings, making them potentially relevant to similar international settings. Moreover, the use of well-established and validated psychometric instruments lends credibility and reliability to the results. The study informs EMS leadership of the impact of stressors on paramedics. Further, our findings may inform individuals wanting to join the paramedic profession so that they can prepare themselves accordingly.

As with many surveys, there is a risk that participants responded in ways they believed were socially or organisationally acceptable, not fully reflecting their actual experiences and practices. Additionally, the online nature of the survey, while efficient, limited the opportunity to explore participants’ perspectives in greater depth, something richer qualitative interviews might have offered. Finally, while the study provides important insights into the experiences of HMCAS paramedics, the findings may not be directly transferable to other contexts with different healthcare systems or cultural dynamics, less well-funded, or where the pandemic was handled differently or with a higher mortality rate.

## 5. CONCLUSION

The COVID-19 pandemic was associated with a significant decline in adaptive coping strategies among HMCAS paramedics. Reductions in social engagement, physical activity, and religious practices were observed, alongside increased reliance on maladaptive coping behaviours. Sociodemographic and occupational factors, including age, education level, and career stage, influenced coping patterns to varying degrees. Religious coping demonstrated both protective and risk-related effects within this context. Overall, these findings highlight the substantial psychological burden experienced by paramedics during the pandemic and underscore the importance of sustained mental health support.

## ACKNOWLEDGEMENT

The authors are thankful to the staff from Hamad Medical Corporation Ambulance Service for their participation in this study and their dedication to helping patients during the COVID-19 pandemic.

## AUTHORS’ CONTRIBUTION

MA: Conceptualization, Methodology, Literature review, Data collection tool design, Data collection, Data analysis, Data interpretation, Writing – original draft, Final manuscript approval. PG: Critical revisions, Data collection, Manuscript review and editing, and Final manuscript approval. HA: Literature review, Data validation, Manuscript review and editing, Final manuscript approval. GK: Critical revisions, Manuscript review and editing, Final manuscript approval. JL: Data interpretation, Manuscript review and editing, Final manuscript approval. GA: Supervision, Conceptualization, Literature review, Data collection tool design, Data collection, Data interpretation, Article writing, Final manuscript approval.

## CONFLICT OF INTEREST

The authors declare no conflict of interest.

## DISCLOSURE OF AI USE

The authors only used Microsoft Copilot as a proofreading tool of the final manuscript before submission to improve the grammar. Copilot was not used to generate the research ideas, study design, methodology, results, or substantive scientific content of the manuscript.

## DATA AVAILABILITY

Aggregated data are available upon reasonable request from the corresponding author.

## FUNDING

The author(s) received no financial support for the research, authorship, and/or publication of this article.

## ETHICAL APPROVAL

Ethical approval was obtained from the Doha Institute for Graduate Studies Institutional Review Board (DI-IRB-2020-S84) and Hamad Medical Corporation Medical Research Center (MRC-01-20-1156).

## Figures and Tables

**Table 1.** Demographic characteristics of the study participants.

Baseline characteristics		Frequency	Percentage (%)
1- Scope of practice?	Ambulance paramedic	255	93.1
	Critical care paramedic	17	6.2
2- Gender?	Female	14	5.1
	Male	258	94.9
3- Ethnicity?	Arabian (West Asia and North Africa)	149	54.8
	Southeast Asian (Philippines…)	60	22.1
	South Asian (India, Sri Lanka …)	45	16.5
	Caucasian	13	4.8
	Black/African	5	1.8
4- Nationality?	Tunisian	74	27.2
	Jordanian	70	25.7
	Filipino	63	23.2
	Indian	42	15.4
	South African	15	5.5
	Other	2	0.7
	British	1	0.4
	Egyptian	1	0.4
	Irish	1	0.4
	Palestinian	1	0.4
	Somalia	1	0.4
	Missing	1	0.4
5- Age?	21–30 years old	19	7.0
	31–40 years old	185	68.0
	41–50 years old	55	20.2
	51–60 years old	13	4.8
6- Duration of employment with HMCAS?	1–3 years	46	16.9
	4–6 years	59	21.7
	7–10 years	106	39.0
	More than 10 years	61	22.4
7- Number of years of experience as a clinician?	5 years or less	26	9.6
	6–10 years	109	40.1
	11–15 years	90	33.1
	More than 15 years	47	17.3
8- Highest educational qualification?	Diploma or less	12	4.4
	Bachelor	206	75.7
	Higher diploma	25	9.2
	Masters	17	6.3
	PhD or equivalent	12	4.4
9- Marital status	Married	240	88.2
	Single	30	11.0
	Divorced or separated	2	0.7
10- Spouse in Qatar (If applicable)	No	24	9.9
	Yes	218	90.1
11- Tested positive for COVID-19 during the pandemic?	Yes	68	25.0
	No	204	75.0

**Table 2.** Coping strategies used by paramedics before and during the pandemic.

Items	Mean ± SD on a 5-point scale (*n* = 272)		Mean Differences	95% Confidence Interval of the Difference		*t*-value	*p*-value
	Before COVID-19	During COVID-19		Lower	Upper		
Use meditation	2.04 ± 1.13	2.21 ± 1.23	0.16	0.09	0.24	4.248	<0.001*
Use comfort food/eat more	2.78 ± 1.10	2.81 ± 1.19	0.03	−0.05	0.12	0.763	0.446
Go out to the cinema	2.16 ± 1.12	1.38 ± 0.78	−0.78	−0.92	−0.65	−11.427	<0.001*
Watch movies/TV	3.10 ± 1.09	2.94 ± 1.21	−0.16	−0.26	−0.05	−2.960	0.003
Browse social media	3.66 ± 0.97	3.67 ± 1.05	0.01	−0.08	0.09	0.165	0.869
Practice religious rituals	3.54 ± 1.18	3.42 ± 1.24	−0.13	−0.21	−0.04	−3.003	0.003
Go for a walk	3.28 ± 1.02	2.78 ± 1.1	−0.50	−0.62	−0.38	−8.292	<0.001*
Spend time alone	2.75 ± 1.01	2.96 ± 1.12	0.21	0.12	0.29	4.557	<0.001*
Spending time with friends	3.16 ± 0.97	2.39 ± 0.92	−0.77	−0.89	−0.65	−12.548	<0.001*
Spending time with family	3.83 ± 1.06	3.42 ± 1.21	−0.41	−0.51	−0.31	−7.961	<0.001*
Reflect on the work situation bothering me	2.81 ± 1.07	3.00 ± 1.12	0.19	0.11	0.28	4.450	<0.001*
Talk with someone about the work situation bothering me	2.76 ± 1.11	2.87 ± 1.18	0.10	0.02	0.19	2.404	0.017
Practice a team sport	2.57 ± 1.30	1.83 ± 1.06	−0.75	−0.88	−0.61	−10.747	<0.001*
Practice a solitary sport	3.35 ± 1.18	2.42 ± 1.30	−0.93	−0.96	−0.90	−60.071	<0.001*
Smoke cigarettes or Shisha	1.78 ± 1.18	1.85 ± 1.34	0.08	−0.01	0.16	1.751	0.081
Use of alcohol or recreational drugs	1.74 ± 1.20	1.72 ± 1.19	−0.02	−0.13	0.10	−0.282	0.779
Total/80	48.96 ± 6.71	45.18 ± 6.66	−3.78	−4.28	−3.28	−14.906	*p* < 0.001*

*Significant at *p* ≤ 0.05; *p* > 0.05: Not significant; n: Number; t: Paired *t*-test.

**Table 3.** Additional coping strategies reported by participants during the pandemic.

Other coping strategies	
Enjoying life	Sleeping for a long time
Going out with friends	Playing cards
Socializing with friends	Practising a sport
Going to the gym	Swimming
Drinking wine, liquor	Drinking juice
Vaping	Staring in empty space
Painting	Resting during the shift
Working a 12-hour shift (extended shift)	

**Table 4.** Changes in paramedics’ coping levels before and during the pandemic (Adapted from Brief COPE scale.^15^

Groups	Coping score (*n* = 272)		McNemar-Bowker test	*p*-value
	Before COVID-19	During COVID-19		
	*n* (%)	*n* (%)		
Low level of coping (17–29)	4 (1.5%)	6 (2.2%)	54.563	<0.001*
Moderate level of coping (30–44)	61 (22.4%)	118 (43.4%)		
High level of coping (45–85)	207 (76.1%)	148 (54.4%)		
Total	272 (100%)	272 (100%)		

*Significant at *p* ≤ 0.05; *p* > 0.05: Not significant; n: Number; SD: Standard deviation; £: Paired *t* test; €: Independent *t* test; F: ANOVA test; Maximum score = 85.

**Table 5.** Association between paramedics’ clinical role, gender, ethnicity, nationality, age, employment, duration in the ambulance service, and coping scores before and during the pandemic.

Scope of practice	(*n* = 272)	Mean coping score ± SD		Statistical test^£^		
		Before COVID-19	During COVID-19	Mean Diff	*t*-value	*p*-value
All participants	272	48.96 ± 6.71	45.18 ± 6.66	−3.78	−14.906	<0.001*
Ambulance paramedic	255	49.08 ± 6.77	45.09 ± 6.66	−3.984	−15.813	<0.001*
Critical care paramedic	17	47.24 ± 5.55	46.47 ± 6.80	−0.765	−0.581	0.569
Statistical test^€^	t^€^	−0.824	1.097			
	*p*-value	0.410	0.273			
**Gender**						
Female	14	52.29 ± 1.76	49.07 ± 1.79	−3.21	−3.342	0.005*
Male	258	48.78 ± 0.42	44.97 ± 0.41	−3.81	−14.520	<0.001*
Statistical test^€^	t^€^	1.912	2.261			
	*p*-value	0.057	0.025*			
**Ethnicity**						
Arabian (West Asia and North Africa)a	149	48.48 ± 0.59	45.25 ± 0.57	−3.23	−9.591	< 0.001*
Black/Africanb	5	49.20 ± 2.6	43.00 ± 3.74	−6.20	−1.673	0.170
Caucasianc	13	52.23 ± 1.89	47.46 ± 1.29	−4.77	−3.318	0.006*
South Asian (Indian …)d	45	48.71 ± 1.02	45.36 ± 1.02	−3.36	−8.887	< 0.001*
Southeast Asian (Filippino…)e	60	49.63 ± 0.69	44.57 ± 0.76	−5.07	−8.792	< 0.001*
Statistical test^€^	F	1.137	0.651			
	*p*-value	0.340	0.627			
**Nationality**						
Filipino	63	50.13 ± 0.82	46.32 ± 0.80	−3.81	−9.722	< 0.001*
Indian	42	50.05 ± 0.92	45.12 ± 1.09	−4.93	−6.647	< 0.001*
Jordanian	70	47.54 ± 0.89	44.04 ± 0.84	−3.50	−7.012	< 0.001*
South African	15	46.93 ± 1.48	45.40 ± 1.62	−1.53	−1.358	0.196
Tunisian	74	49.34 ± 0.78	45.11 ± 0.75	−4.23	−8.803	< 0.001*
Other	8	46.88 ± 1.30	46.75 ± 2.19	−0.125	−0.062	0.952
Statistical test^€^	F	1.725	0.868			
	*p*-value	0.129	0.503			
**Age (years)**						
21–30 (a)	19	49.16 ± 1.42	46.42 ± 1.53	−2.74	−3.980	< 0.001*
31–40 (b)	185	49.56 ± 0.47	45.38 ± 0.47	−4.18	−12.582	< 0.001*
41–50 (c)	55	48.62 ± 0.86	45.40 ± 0.86	−3.22	−6.769	< 0.001*
51–60 (d)	13	41.62 ± 2.41	39.62 ± 2.48	−2.00	−3.854	0.002*
Statistical test^€^	F	6.067	3.407			
	*p*-value	0.001	0.018*			
Post hoc test (*p*-value)	a vs. b	0.797	0.511			
	a vs. c	0.756	0.560			
	a vs. d	0.001*	0.004*			
	b vs. c	0.347	0.983			
	b vs. d	0.001*	0.002*			
	c vs. d	0.001*	0.005*			
**Number of years of employment at HMCAS**						
1–3 years	46	50.28 ± 1.04	46.67 ± 1.09	−3.61	−5.809	<0.001*
4–6 years	59	49.15 ± 0.96	45.51 ± 0.92	−3.64	−7.061	<0.001*
7–10 years	106	48.42 ± 0.59	44.55 ± 0.61	−3.87	−8.708	<0.001*
More than 10 years	61	48.74 ± 0.88	44.84 ± 0.79	−3.90	−8.157	<0.001*
Statistical test^€^	F	0.867	1.195			
	*p*-value	0.459	0.312			

*Significant at *p* ≤ 0.05; *p* > 0.05: Not significant; n: Number; SD: Standard deviation; £: Paired *t* test; €: Independent *t* test; F: ANOVA test; Maximum score = 85.

**Table 6.** Association between paramedics’ years of clinical experience, level of education, marital status, spouse location, and COVID-19 test results, and coping scores before and during the pandemic.

Years of Clinical Experience	(*n* = 272)	Mean coping score ± SD		Statistical test^£^			
		Before COVID-19	During COVID-19	Mean Diff		*t*-value	*p*-value
5 years or less	26	51.15 ± 1.42	48.12 ± 1.63	−3.04		−3.496	0.002
6–10 years	109	48.22 ± 0.61	44.61 ± 0.62	−3.61		−8.600	<0.001*
11–15 years	90	49.32 ± 0.68	44.88 ± 0.62	−4.44		−10.577	<0.001*
More than 15 years	47	48.79 ± 1.1	45.47 ± 1.06	−3.32		−5.849	<0.001*
Statistical test^€^	F	1.475	2.069				
	*p*-value	0.222	0.105				
**Educational qualification**							
Diploma or less (a)	12	44.50 ± 2.49	41.42 ± 2.48	−3.08		−2.846	0.016*
Bachelor (b)	206	48.91 ± 0.47	44.90 ± 0.45	−4.01		−13.665	<0.001*
Higher Diploma (c)	25	50.28 ± 1.31	46.80 ± 1.47	−3.48		−4.809	<0.001*
Master’s or PhD (d)	29	50.07 ± 1.03	47.34 ± 1.17	−2.72		−3.191	0.003*
Statistical test^€^	F	2.396	2.978				
	*p*-value	0.069	0.032*				
Post hoc test (*p*-value)	a vs. b		0.076				
	a vs. c		0.021*				
	a vs. d		0.009*				
	b vs. c		0.174				
	b vs. d		0.062				
	c vs. d		0.762				
**Marital status**							
Married	240	49.05 ± 0.42	45.24 ± 0.42	−3.81	−13.914		<0.001*
Single	30	48.23 ± 1.46	45.00 ± 1.45	−3.23	−5.003		<0.001*
Divorced or separated	2	49.00 ± 2.00	40.50 ± 5.50	−8.50	−2.429		0.249
Statistical test^€^	F	0.199	0.513				
	*p*-value	0.820	0.599				
**Location of spouse**							
Spouse in Qatar	218	49.75 ± 1.06	47.13 ± 1.33	−2.63		−2.355	0.027*
Not Applicable (NA)	30	49.1 ± 1.22	45.4 ± 1.31	−3.70		−5.298	<0.001*
Spouse outside Qatar	24	48.86 ± 0.47	44.94 ± 0.45	−3.92		−14.214	<0.001*
Statistical test^€^	F	0.197	1.188				
	*p*-value	0.821	0.307				
**Positive test for COVID-19 during the pandemic**							
Yes	68	49.54 ± 8.58	45.65 ± 8.33	−3.897		−7.894	<0.001*
No	204	48.77 ± 5.97	45.02 ± 6.02	−3.745		−12.638	<0.001*
Statistical test^€^	t^€^	0.667	0.824				
	*p*-value	0.505	0.411				

*Significant at *p* ≤ 0.05; *p* > 0.05: Not significant; n: Number; t: Paired *t*-test.
